# ﻿Two new ant species of the genus *Leptogenys* (Hymenoptera, Formicidae) from Hainan, China, with a key to the known Chinese species

**DOI:** 10.3897/zookeys.1195.115889

**Published:** 2024-03-15

**Authors:** Chao Chen, Zhilin Chen, Zhenghui Xu, Qishan Fu, Liwen Fu

**Affiliations:** 1 Kunming Natural History Museum of Zoology, Kunming Institute of Zoology, Chinese Academy of Sciences, Kunming, Yunnan 650221, China Kunming Institute of Zoology, Chinese Academy of Sciences Kunming China; 2 Key Laboratory of Ecology of Rare and Endangered Species and Environmental Protection, Guangxi Normal University, Ministry of Education, Guilin, 541004, China Guangxi Normal University Guilin China; 3 Key Laboratory of Forest Disaster Warning and Control in Yunnan Province, College of Biodiversity Conservation, Southwest Forestry University, Kunming, Yunnan Province 650224, China Southwest Forestry University Kunming China; 4 Yinggeling Branch of Hainan Tropical Rainforest National Park Administration, Baisha, 572800, China Yinggeling Branch of Hainan Tropical Rainforest National Park Administration Baisha China

**Keywords:** *L.crassicornis* species group, *L.leleji* species group, Ponerinae, southern China, taxonomy

## Abstract

Two new species of ponerine ants from Hainan Province, China, *Leptogenyshainanensis***sp. nov.** and *L.zhoui***sp. nov.**, are delineated and depicted based on the worker caste. *Leptogenyshainanensis***sp. nov.** belongs to the *L.leleji* species group, with mandibles elongate, slender and curved, lacking a distinct masticatory margin. On the other hand, *L.zhoui***sp. nov.** belongs to the *L.crassicornis* species group, distinguished by its square head, smooth body, mandibles with a dentate masticatory margin, and short antennae. A key to workers for the known species of *Leptogenys* in China are provided and a map is provided for the newly described species.

## ﻿Introduction

The genus *Leptogenys* Roger, 1861, encompasses more than 316 valid species and 14 valid subspecies (Bolton 2023). Twenty-four species of the genus *Leptogenys* are recorded in China. Predominately tropical and subtropical, there are some species that could be argued that extended into temperate, more seasonal regions (Guйnard et al. 2017). Nests of New World *Leptogenys* may vary from 20 to 30 workers, rarely surpassing 50. Small nest sizes are also reported for more than 15 species of this genus in the Oriental Tropics by Ito and Ohkawara (2000) with the outstanding exception of some SE Asian *Leptogenys* with army ant habits, which have colonies numbering in the thousands, and are generalists, taking diverse prey (Witte and Maschwitz 2002). These ants are known for their preference for small colonies and some have specialized predation on isopods (Schmidt and Shattuck 2014) or earwigs (Steghaus-Kovac and Maschwitz 1993) for Oriental species, and termite predation for the African *nitida* group (Bolton 1975). In Cambodia and Thailand, *L.cyanicatena* Arimoto & Yamane specialize on a broad range of millipedes, and workers link together in chains to retrieve the larger millipedes.

The features of *Leptogenysleleji* Zryanin, including the shape of its cranio-mandibular system and petiole, bear resemblance to those of the representatives of the *L.conradti* species group (CASENT0907340) from Africa, the *L.turneri* species group (*L.longensis*CASENT0217531) from Australia (Zryanin 2016), and certain species of the *L.unistimulosa* species group (CASENT0178818) from the New World (Lattke 2011). On the other hand, the *L.crassicornis* species group (CASENT0281924) is distinguished by its square-shaped head, smooth body, mandibles with a dentate masticatory margin, and short antennae (Xu 2000).

Significant contributions to the understanding of *Leptogenys* on a global scale have been made through species listings, revisionary efforts, and the diagnostic and synoptic description of the genus by various researchers, including Lattke (2011), Sarnat and Economo (2012), Zhou et al. (2012), Bharti and Wachkoo (2013), Rakotonirina and Fisher (2014), Schmidt and Shattuck (2014), Xu and He (2015), Zryanin (2016), Sharaf et al. (2017), Arimoto (2017a, 2017b), Arimoto and Yamane (2018), Lуpez-Muсoz et al. (2018), Wachkoo et al. (2018), Ramage et al. (2019), Fernбndez and Guerrero (2019), and Subedi et al. (2022). Considerable effort is still required to unravel the regional diversity of *Leptogenys* species. A contemporary revision of Asian *Leptogenys* is notably absent.

In China, the first record of four *Leptogenys* species were documented by Wheeler (1930), followed by subsequent reports of Chinese species by Tang et al. (1995) (five species), Wu and Wang (1995) (three species), Xu (1996) (seven species), Xu (2000) (13 species), Zhou (2001) (seven species from Guangxi), Xu (2002) (11 species from Yunnan), Wang et al. (2009) (two species from Hubei), and Terayama (1990, 2009) (four species from Taiwan). Guйnard and Dunn (2012) listed 20 species in China, while Xu and He (2015) revised the Oriental species and recorded 24 species in China.

The present study contributes to this body of work by describing *Leptogenyshainanensis* sp. nov. and *L.zhoui* sp. nov. from Hainan Province, China, accompanied by high-resolution images and measurements of important morphological characters. A key to all known Chinese species and a map of the distribution data of the two new species is provided.

## ﻿Materials and methods

The ant specimens were obtained using sample-plot and search-collecting methods (e.g., Xu 2002). Subsequently, the specimens were meticulously examined using an SDPTOP-SZM stereomicroscope. High-quality multifocus montage images were captured using a Keyence VHX-6000 ultra-depth microscopic three-dimensional microscope. The map was constructed using the software package ArcGIS v. 10.8. To compare the worker morphology of the two new species, reference was made to the original descriptions of related species (Emery 1887, 1895; Forel 1900, 1915; Zryanin 2016). sculptural, and hair terminology follow Harris (1979) and Wilson (1955). Images of *L.crassicornis* species group (CASENT0281924) were sourced from AntWeb (2023).

Standard measurements and indices were employed as defined in Bolton (1975) and Lattke (2011), with the addition of ML and ED, as outlined below. Furthermore, alitrunk length is substituted by WL (Weber’s Length) in accordance with the methodology proposed by Xu and He (2015). Addition of HLL, HLA, CML, PrL, and PrH (Arimoto 2017a). All measurements are reported in millimeters.

**HL** Head length: straight-line length of head in perfect full-face view, measured from the mid-point of the anterior clypeal margin to the midpoint of the posterior margin. In species where one or both of these margins are concave, the measurement is taken from the mid-point of a transverse line that spans the apices of the projecting portions.

**HLL** Head lateral margin length: in full-face view, the head length measured from the mandible base to the nuchal carina.

**HLA** Anterior head length: in full-face view, the head length measured from the mandible base to the anterior edge of the eye.

**HW** Head width: maximum width of head in full-face view, excluding the eyes.

**ML** Mandible length: straight-line length of mandible measured from apex to the lateral base.

**CML** Clypeal median lobe length: in full-face view, the straight-line length measured from the anterior margin of the clypeus to the anterior margin of the torulus.

**CI** Cephalic Index = HW × 100 / HL.

**SL** Scape length: straight-line length of the antennal scape, excluding the basal constriction or neck.

**SI** Scape index = SL × 100 / HW.

**ED** Eye diameter: maximum diameter of eye.

**PrL** Pronotum length: in profile, the diagonal length of the pronotum, measured from the anterior margin of the pronotum excluding the collar to the posterior extremity of the pronotum.

**PrH** Pronotum height: in profile, the maximum height of the pronotum, measured from the posterior base of the lateral margin of the pronotum to the highest point of the pronotum.

**PrW** pronotum width: maximum width of pronotum measured in dorsal view.

**WL** Weber’s length (= alitrunk length): diagonal length of the mesosoma in lateral view, measured from the point at which the pronotum meets the cervical shield to the posterior basal angle of the metapleuron.

**TL** Total length: total outstretched length of the individual, from the mandibular apex to the gastral apex.

**PL** Petiole length: length of petiole measured in lateral view from the anterior process to the posteriormost point of the tergite, where it surrounds the gastral articulation.

**PH** Petiole height: height of petiole measured in lateral view from the apex of the ventral (subpetiolar) process vertically to a line intersecting the dorsal most point of the node.

**DPW** Dorsal petiole width: maximum width of petiole in dorsal view.

**LPI** Lateral petiole index = PH × 100 / PL.

**PDPI** Dorsal petiole index = DPW × 100 / PL.

## ﻿Taxonomic account

### ﻿ Leptogenys hainanensis sp. nov.

Taxon classificationAnimalia

56CACF68-5C14-5A3F-BDBA-FE2A9D904FD8

https://zoobank.org/39DED636-393B-4EA7-B0E7-ABD4220B94A3

Figs 1A–D , 2

#### Type material.

***Holotype***: worker, China: Hainan Province, Qiongzhong County, Yinggeling Nature Reserve, Yinggezui sub-station, 19.048333°N, 109.559167°E, 750 m, 28.VII.2022, Chao Chen leg. The holotype specimen is deposited in Kunming Natural History Museum of Zoology, Kunming Institute of Zoology, Chinese Academy of Sciences (KIZCAS), Kunming, Yunnan Province, China, Reg. No. KIZ20220009 (unique specimen identifiers). ***Paratype***: 1 worker, China: Hainan Province, Ledong County, Jianfengling, 18.727222N, 108.898611E, 950 m, 9.IV.2016, Zhi-Lin Chen leg. The paratype specimen is deposited in the Insect Collection, Guangxi Normal University (GXNU), Guilin, Guangxi Zhuang Autonomous Region, China, Reg. No. G160246 (unique specimen identifiers).

#### Description.

**Figure 1.**
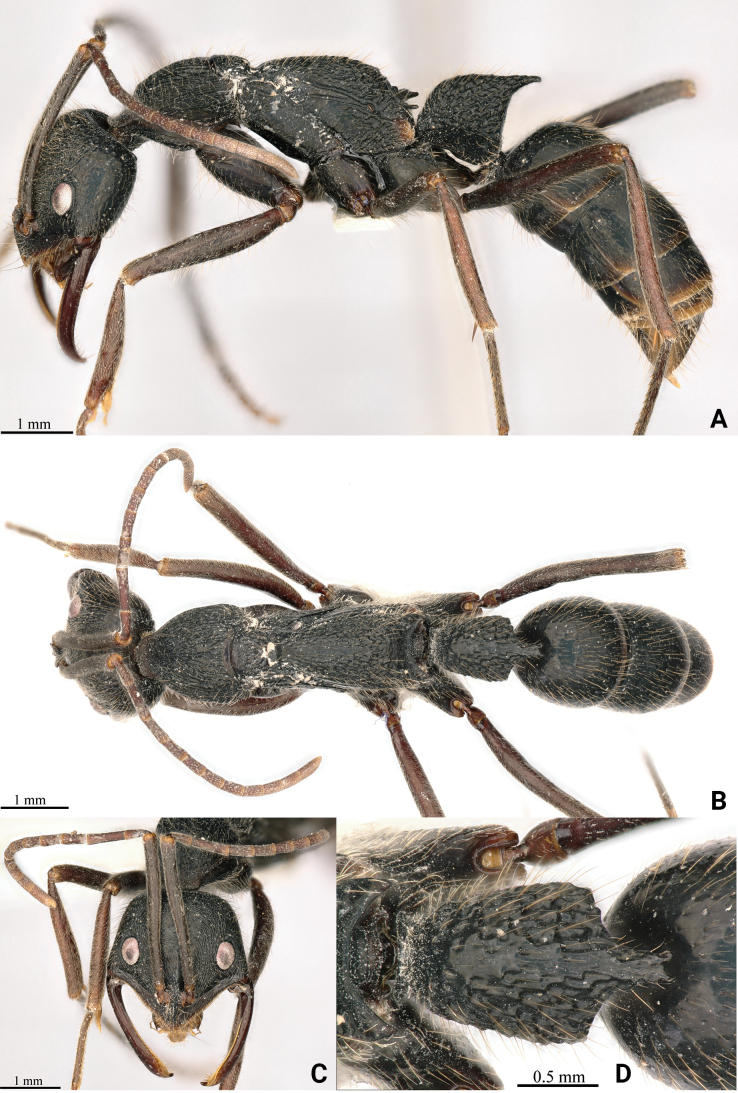
*Leptogenyshainanensis* sp. nov. worker **A** body in lateral view **B** body in dorsal view **C** head in full-face view **D** petiole in dorsal view.

**Holotype worker** (Fig. 1A–D): HL 2.34, HLL 1.47, HLA 0.33, HW 2.42, ML 1.87, CML 0.45, CI 103, SL 2.54, SI 105, ED 0.48, PrL 1.44, PrH 1.06, PrW 1.58, WL 4.23, TL 13.1, PL 1.22, PH 1.54, DPW 1.05, LPI 126, PDPI 86.

In full-face view, head presents a roughly trapezoidal shape, with anterior widening, length slightly longer than its width. Posterior margin straight and carinate, posterior corners narrowly rounded, lateral margins display a subtle convexity. Mandibles elongate and slender, with a masticatory margin, forming into the inner margin without a distinct angle; lamellae extension at masticatory margin and inner mandible; large gap formed between clypeus and mandible when fully closed (Fig. 1C). Clypeus triangularly produced, with blunt apex; anterior clypeal margin fringed with narrow translucent lamella; median longitudinal carina of clypeus sharp. Antennae composed of 12 segments, with scape extending beyond posterior corner of head by 2/5 of its length (SI 105). Basal flagellar (third antennal) segment elongate, markedly longer than neighboring antennal segments (lengths of segments 2, 3, 4: 0.36 mm, 0.64 mm, 0.43 mm). Eyes moderately large, occupying ~ 1/3 of lateral cephalic margin, position in close proximity to base of mandibles (HLA 0.33).

**Figure 2.**
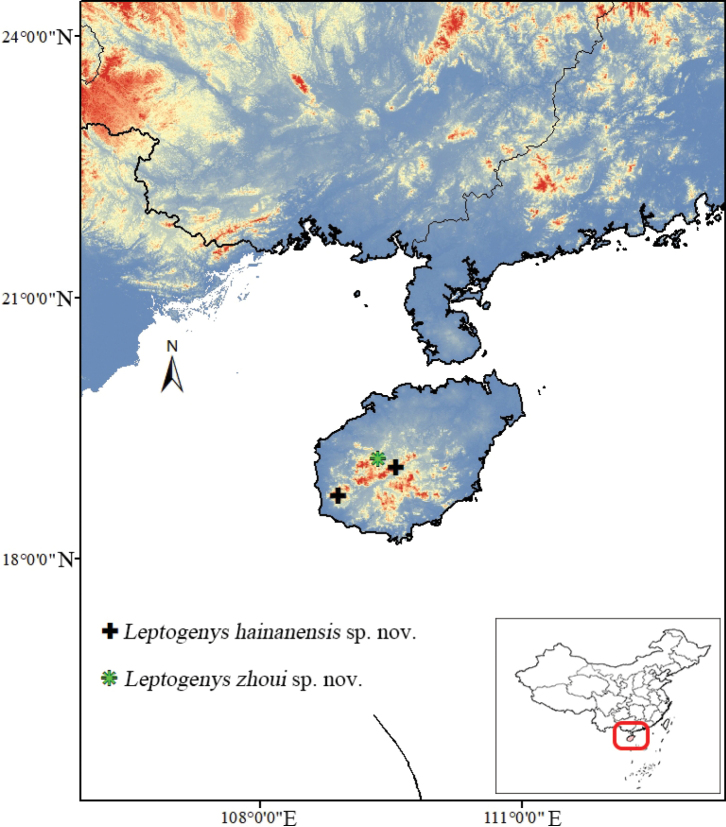
Distribution of the two new species of *Leptogenys* in Hainan, China. The gradients of the map indicate the topography.

In lateral view, promesonotum presents a moderately convex form and a slight elevation compared to propodeum. Promesonotal suture is discernible but not deeply marked. Prosternal process blunt round. Metanotal groove narrow and deeply impressed. Dorsum of propodeum displays a gentle convexity and slopes posteriorly, with a length ~ 2 × that of the declivity. Propodeum armed with triangular tooth at level of spiracle; propodeal declivity with carina uniting apices of these teeth; area anteriad to carina concave in cross-section, with mostly smooth and shiny surface; area posteriad to carina depressed as broad transverse sulcus. Petiolar node robust and approximately trapezoidal in shape, displaying a slight posterior inclination. Its anterior and dorsal margins exhibit a mild convexity, with anterodorsal corner broadly rounded and posterior margin straight. An acute spine extends posterodorsally from posterodorsal corner. Subpetiolar process shaped as sub-rectangular tubercle with gentle posterior slope. Gaster roughly cylindrical, with distinct constriction between two basal segments and extruding sting. Prora shaped as sharp, ventrally directed, lobe at anteroventral angle of first gastral segment.

In dorsal view, mesosoma exhibits a slight constriction at mesopleuron, with pronotum being marginally wider than propodeum. Sides of pronotum feature moderate convexity. Promesonotal suture and deeply notched metanotal groove present. Sides of mesopleuron appear almost straight, while those of propodeum display a mild convexity. Petiolar node roughly trapezoidal, widening posteriorly, with slightly convex sides and posterior margin that extends into triangular process with bifid apex.

Mandibles finely longitudinally striated with smooth and shiny interspaces, while clypeus and dorsum of the head exhibit dense longitudinal striae, vertex displaying dense transverse striae. Mesosomal dorsum densely longitudinally striated, with striation transitioning to reticulate pattern on mesonotum and posterior part of propodeum. Mesopleura and metapleura relatively smooth, with few oblique striations on lower part of metapleura. Petiole coarsely reticulate, with short, small prominences at interface. Gastral have many distinct hairs bearing punctures. Abundant suberect to subdecumbent short hairs and depressed pubescence adorn dorsal aspect of body, appressed pubescence present on cephalic dorsum, antennae, and legs. Scapes and tibiae exhibit dense depressed pubescence. Body displays black coloration, with mandibles, apical antennal segments, and legs exhibiting reddish brown hue, and eyes appearing grey.

**Paratype worker**: HL 2.11, HLL 1.35, HLA 0.29, HW 2.17, ML 1.92, CML 0.39, CI 102, SL 2.40, SI 111, ED 0.48, PrL 1.51, PrH 1.01, PrW 1.62, WL 4.08, TL 12.6, PL 1.26, PH 1.46, DPW 1.03, LPI 116, PDPI 82 (*n* = 1). Resembling the holotype worker, the specimen exhibits relatively coarser striation on the mesosomal dorsum and obliquely coarse striation on the metapleura. Additionally, the legs display a brownish black hue.

**Queen and male.** Unknown.

#### Comparative notes.

**Figure 3.**
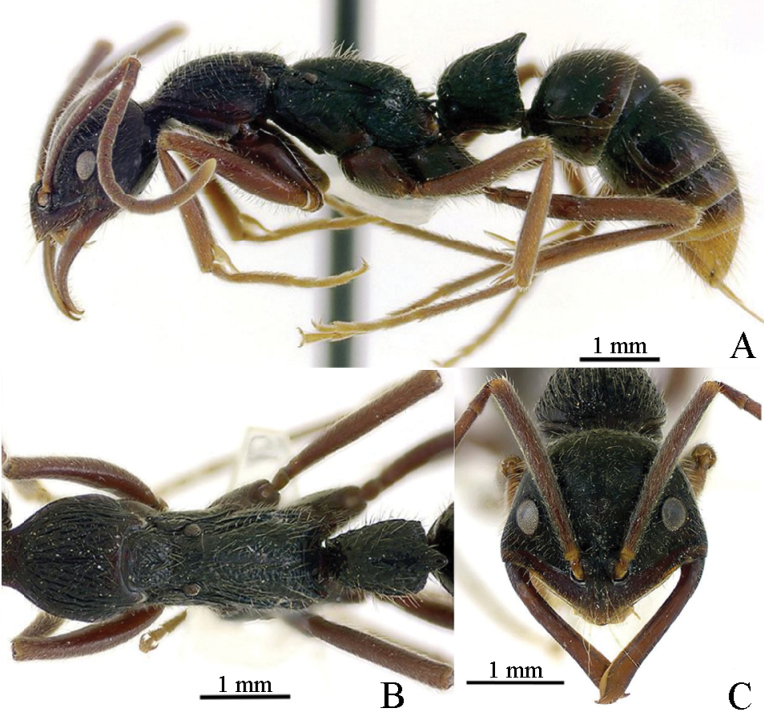
*Leptogenysleleji* worker (from AntWeb 2023, photographer: V.A. Zryanin) **A** body in lateral view **B** mesosoma and petiole in dorsal view **C** head in full-face view.

In addition to being similar to *Leptogenysleleji* Zryanin, 2016, this new species is significantly different from other Chinese and Oriental species. The common characteristics with *L.leleji* (Fig. 3A–C) are as follows: the cephalic capsule is wider than long; the anterior clypeal margin is fringed with a narrow translucent lamella; the mandibles are linear, a large gap is formed between clypeus and mandible when fully closed; the basal flagellar (third antennal) segment is elongate; the dorsum of the body with standing hairs; the propodeum with lateral teeth, and posterior apex of petiole in profile is drawn out into a tooth (Zryanin 2016). In the new species (Fig. 1A–D), with the head in full-face view, the posterior margin is straight and carinate, and the posterior corners are narrowly rounded, while the lateral margins display a subtle convexity; the distance between the ventral eye margin and the anterior clypeal margin is shorter (HLA 0.33); dorsum of the head exhibits dense longitudinal striae; the eyes’ greatest diameter is greater than the maximal width of the scape; the posterior process of the petiolar node is relatively longer and bifid at the apex, with an abundance of short, small prominences on the reticulation interface. Conversely, in *L.leleji*, the head in full-face view is markedly wider anteriorly than posteriorly, the lateral and posterior margins form a continuous convexity, the occipital carina is distinct; the distance between the ventral eye margin and the anterior clypeal margin is moderate (HLA 0.41); dorsum of the head with sparse longitudinal striae; eyes’ greatest diameter is greater than the maximal width of the scape; the posterior process of the petiolar node is relatively shorter and not bifid at the apex, and lacks the short, small prominences on the reticulation interface.

#### Etymology.

The species name *hainanensis* is a Latin feminine singular adjective in the nominative case and refers to Hainan Province, where the first specimen of this species was collected. The name is unchangeable.

### ﻿ Leptogenys zhoui sp. nov.

Taxon classificationAnimalia

2318B587-D1DF-51C3-8115-690EC24FFF86

https://zoobank.org/C5B46D93-7930-4CA0-A1A9-05F3FA8A5EA2

Figs 2 , 4A–D

#### Type material.

***Holotype***: worker, China: Hainan Province, Baisha County, Nanmeiling forest park, Yaqiong sub-station, 19.144167°N, 109.349167°E, 700m, 2.VIII.2022, Chao Chen leg. The holotype specimen is deposited in Kunming Natural History Museum of Zoology, Kunming Institute of Zoology, Chinese Academy of Sciences (KIZCAS), Kunming, Yunnan Province, China, Reg. No. KIZ20220196 (unique specimen identifiers). ***Paratypes***: 2 workers, data the same as holotype. One paratype worker is deposited in GXNU. No. KIZ20220197 (unique specimen identifiers); 1 paratype worker is deposited in the Insect Collection, Southwest Forestry University (SWFU), Kunming, Yunnan Province, China. No. KIZ20220198 (unique specimen identifiers)

#### Description.

**Figure 4.**
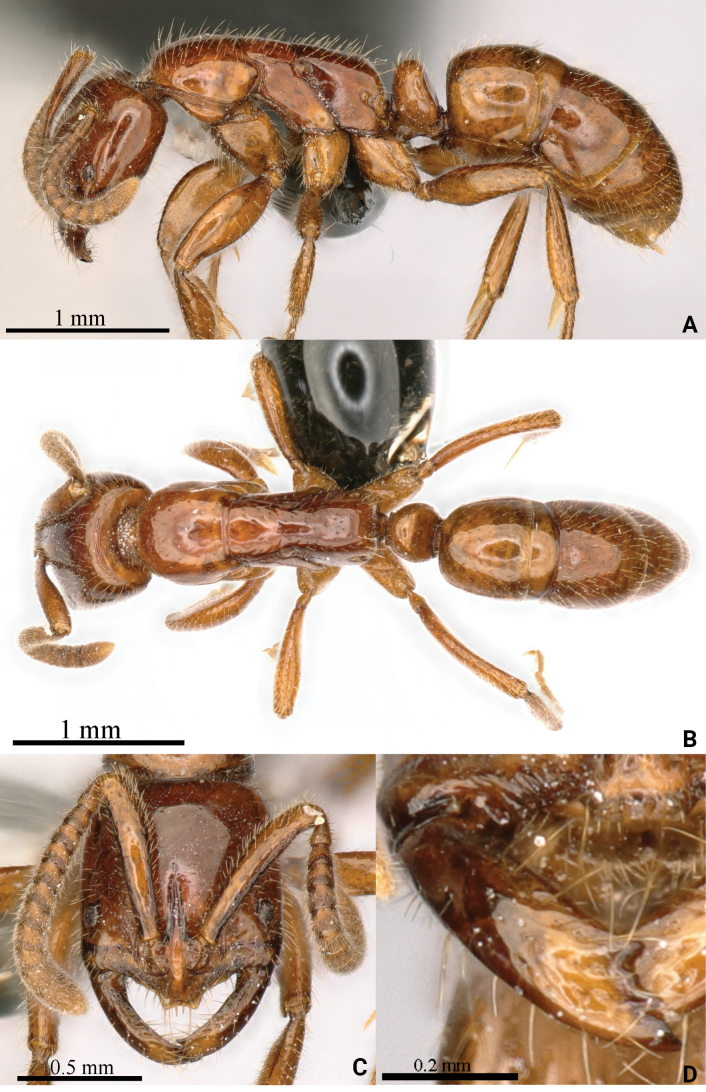
*Leptogenyszhoui* sp. nov. worker **A** body in lateral view **B** body in dorsal view **C** head in full-face view **D** mandibles in full-face view.

**Holotype worker** (Fig. 4): HL 0.91, HLL 0.75, HLA 0.17, HW 0.81, ML 0.49, CML 0.12, CI 89, SL 0.68, SI 84, ED 0.10, PrL 0.69, PrH 0.49, PrW 0.61, WL 1.53, TL 4.4, PL 0.26, PH 0.46, DPW 0.35, LPI 174, PDPI 135.

Head in full-face view slightly wider anteriorly than posteriorly with straight posterior margins and narrowly rounded posterior corners. Lateral margins exhibit a subtle convexity. Mandibles display parallel inner and outer margins, with masticatory margin adorned with three or four teeth. Anterior portion of clypeus concave medially, while dentate protrusions emerge on both sides. Short frontal ridge extends slightly beyond upper end of eyes, and well-developed frontal lobes cover antennal sockets. 12-segmented antennae boast thickened funiculus towards the end, with scape reaching just to posterior head corner. Eyes, of medium size, have maximum diameter ~ 2/3 that of scape and located at 1/5 in front of lateral cephalic margin (HLL 0.75, HLA 0.17).

In lateral view, promesonotum slightly raised, with distinct promesonotal suture and metanotal groove absent. Promesonotum pleural suture and meso-metathoracic pleural suture evident, terminating at metathoracic spiracle. Dorsum of propodeum appears nearly straight, maintaining same horizontal plane as promesonotum, with slightly convex declivity. Length of dorsum of propodeum is ~ 1.5 × that of declivity, with curved transition from slope at back. Petiolar node carries a roughly trapezoidal shape, with weakly convex anterior margin and straight posterior margin, with convex dorsal margins. Well-developed subpetiolar process features a rounded anterior corner. Gaster adopts an approximately cylindrical form, with sting extruding.

In dorsal view, promesonotal suture distinct, while metanotal groove absent. Pronotum anteriorly a round shape, lateral margins convex. Mesonotum trapezoidal, wider in front and narrowing towards back. Propodeum square, with slightly convex lateral margins. Petiolar node dorsum semicircular, with round front and transverse posterior margin, ~ 1.8 × wider than its length (PL 0.26, PH 0.46).

Mandibles exhibit sparse pits along inner edge, while both sides of clypeus display longitudinal striae. Funiculus appears densely punctate, while remainder of body smooth and shiny. Erect or suberect hairs abundant abaxially on body, with only funiculus adorned with dense decumbent pubescence. Body reddish brown.

**Paratype worker**: HL 0.89–0.90, HLL 0.74–0.75, HLA 0.17, HW 0.81–0.83, ML 0.47–0.50, CML 0.11–0.12, CI 91, SL 0.67–0.68, SI 82, ED 0.10, PrL 0.62, PrH 0.46–0.47, PrW 0.60–0.61, WL 1.42–1.48, TL 4.1–4.2, PL 0.26, PH 0.43–0.45, DPW 0.34-0.37, LPI 165-172, PDPI 131-140 (*n* = 2). Similar to holotype worker.

**Queen and male.** Unknown.

#### Comparative notes.

**Figure 5.**
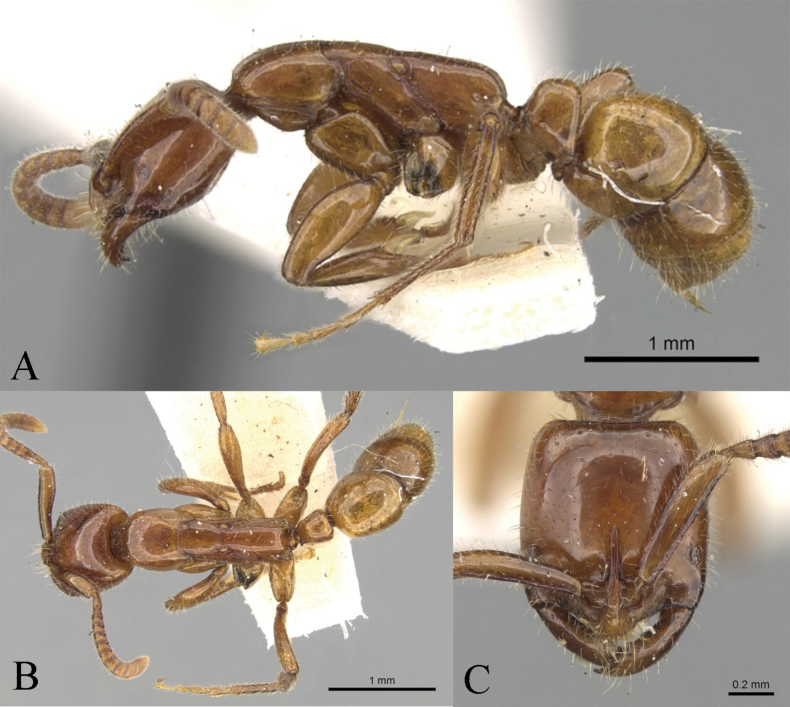
*Leptogenyscrassicornis* worker (from AntWeb 2023, CASENT0281924, photographer: Shannon Hartman) **A** body in lateral view **B** body in dorsal view **C** head in full-face view.

The new species is compared against species within the *L.crassicornis* species group, characterized by a square head, a smooth appearance, mandibles with a dentate masticatory margin, and short antennae. The new species exhibits the closest resemblance to *Leptogenyscrassicornis* Emery, 1895 (Fig. 5A–C). In full-face view of the new species, sparse pits adorn the inner edge of the mandibles, while the middle part of the anterior clypeus displays a concave feature and forms dentate protrusions on both sides. The eyes are of moderate size, with a maximum diameter of ~ 2/3 of the maximum diameter of the scape. In lateral view, the petiolar node is moderately thick and ~ 1/2 the height (PL 0.26, PH 0.46), while the dorsum of the propodeum appears nearly straight and aligns with the promesonotum in the same horizontal plane. Furthermore, the body, abaxially, exhibits a profusion of erect or suberect hairs. Conversely, in *L.crassicornis*, ’the inner edge of the mandible lacks pits or has only one or two pits in full-face view. The anterior aspect of the clypeus is rounded, and the eyes are smaller, with a maximum diameter of ~ 1/2 of the maximum diameter of the scape. In lateral view, the petiolar node appears thick and ~ 4/5 of the height (PL 0.33, PH 0.41), while the dorsum of the promesonotum is higher than the propodeum. Additionally, the body, abaxially, bears sparse erect or suberect hairs and decumbent pubescence.

**Figure 6.**
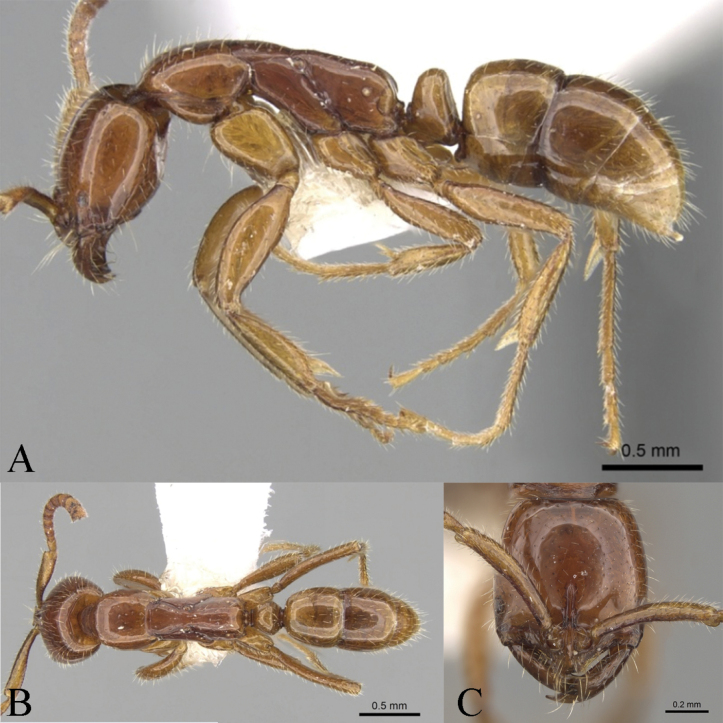
*Leptogenysmyops* worker (from AntWeb 2023, CASENT0281925, photographer: Shannon Hartman) **A** body in lateral view **B** body in dorsal view **C** head in full-face view.

The new species bears resemblance to *Leptogenysmyops* (Emery, 1887) (Fig. 6A–C). In the new species, body size is relatively large (TL > 4 mm); in full-face view, the anterior clypeal margin is fringed with two or three peg-like setae medially; the antennal scape reaches just to the posterior head corner; the eyes are relatively large (ED 0.1 mm); the dorsum of the propodeum appears nearly straight and aligns with the promesonotum in the same horizontal plane. Furthermore, the body, abaxially, exhibits a profusion of erect or suberect hairs. Conversely, in *L.myops*, the body size is relatively small (TL < 4 mm); in full-face view, the anterior clypeal margin is not fringed medially; the antennal scape does not reach the posterior head corner; the eyes are relatively small (ED 0.07 mm); the metanotal groove divides the dorsal outline of the mesosoma into two distinct convexities in lateral view; body, abaxially, bears sparse erect or suberect hairs and decumbent pubescence.

#### Etymology.

The newly discovered species is named in honor of Professor Shanyi Zhou of Guangxi Normal University, in commemoration of his exceptional contributions to the field of ant taxonomy in China. The name *zhoui* was created by adding the singular Latin genitive case suffix -i to the last name of a male person. The name is unchangeable.

### ﻿Key to species of *Leptogenys* found in China based on the worker caste

**Table:** 

1	Masticatory margin of mandible with > 2 teeth and denticles in addition to the apical one	**2**
–	Masticatory margin of mandible usually edentate, at most with 1 tooth in addition to the apical one	**8**
2	Head quadrate or roughly trapezoidal in full-face view approx. as broad as long. Petiolar node longitudinally compressed and squamiform in lateral view, acute at summit, without distinct dorsal margin, the node above anterior and posterior articulations ≥ 2 × as broad as long in dorsal view	**3**
–	Head rectangular in full-face view, distinctly longer than broad. Petiolar node longitudinally thickened or elongate, or roughly triangular, or cubical in lateral view, dorsal margin long or narrowly convex, always distinct, the node above anterior and posterior articulations ≤ 1.5 × as broad as long in dorsal view	**4**
3	Medial side of eyes on dorsum of head longitudinally striate, anteromedian clypeal margin broadly angulate. Antennal segments IV–VI approx. as broad as long. Subpetiolar process broad and triangular. Body color reddish brown (Myanmar, India, China, Thailand)	***L.birmana* Forel**
–	Medial side of eyes on dorsum of head smooth and shiny, anteromedian clypeal margin bidentate. Antennal segments IV–VI longer than broad. Subpetiolar process narrow and cuneiform. Body color brownish black (India, Sri Lanka, Vietnam, China)	***L.processionalis* (Jerdon)**
4	Petiolar node erectly triangular in lateral view, dorsal and anterior margins form a single arch, the two margins not separated by a distinct blunt angle (Vietnam, China)	***L.davydovi* Karavaiev**
–	Petiolar node subquadrate in lateral view, dorsal and anterior margins not forming a single arch, the 2 margins separated by a distinct blunt angle	**5**
5	Petiolar node thickly trapezoidal in lateral view, dorsal margin as long as or longer than anterior margin. Body color brownish yellow (Myanmar, China)	***L.crassicornis* Emery**
–	Petiolar node thinly trapezoidal in lateral view, dorsal margin distinctly shorter than anterior margin. Body color reddish brown to black	**6**
6	Mandible < 5 teeth, dorsum of the propodeum appears nearly straight and aligns with the promesonotum in the same horizontal plane	***L.zhoui* sp. nov.**
–	Mandible ≥ 5 teeth, the dorsum of the promesonotum is higher than the propodeum	**7**
7	Body size is relatively large (TL 6.4 mm). Subpetiolar process semicircular, rounded at apex. Body color reddish brown (China: Guangxi)	***L.strena* Zhou**
–	Body size is relatively small (TL 5.2 mm). Subpetiolar process triangular, angled at apex. Body color black (India, Myanmar; China: Yunnan)	***L.lucidula* Emery**
8	Head dorsum striate throughout	**9**
–	Head dorsum punctate throughout or mostly smooth and shiny, at most punctate or rugose anterior to eyes	**11**
9	Head slightly wider than long in full-face view. Petiolar node posterodorsal corner prominent with elongated triangular denticle	***L.hainanensis* sp. nov.**
–	Head longer than wide in full-face view. Petiolar node posterodorsal corner broadly rounded without triangular denticle	**10**
10	Clypeus with distinct longitudinal central carina. Sides of pronotum smooth, finely rugulose or finely reticulate; sides of mesothorax, metathorax and propodeum irregularly rugose (India, Sri Lanka, Myanmar, China, Thailand, Philippines, Malaysia, Singapore, Indonesia, New Guinea, Solomon Islands	***L.diminuta* (Smith)**
–	Clypeus without longitudinal central carina. Mesosoma and petiolar node regularly longitudinally striate (India, Myanmar, Thailand, Vietnam, China, Malaysia, Indonesia, New Guinea)	. ***L.kitteli* (Mayr)**
11	Head dorsum punctate throughout	**12**
–	Head dorsum mostly smooth and shiny, at most punctate or rugose anterior to eyes	**20**
12	First gastral segment mostly punctate	**13**
–	First gastral segment smooth and shiny	**16**
13	Head roughly rectangular in full-face view, not widening anteriorly, posterior margin roundly convex. Anterior apex of clypeus strongly convex (China)	***L.hezhouensis* Zhou**
–	Head roughly trapezoidal in full-face view, distinctly widening anteriorly, posterior margin nearly straight. Anterior apex of clypeus truncate or nearly truncate	**14**
14	Antennae relatively shorter, only one fourth of length of scape surpassing posterior head corner, segments 3 and 4 approx. equal. Anterior margin of petiolar node in lateral view straight and vertical (China)	***L.yandii* Xu & He**
–	Antennae relatively longer, 1/3 to 1/2 of length of scape surpassing posterior head corner, segment 3 distinctly longer than segment 4. Anterior margin of petiolar node weakly convex and slope, anterodorsal corner broadly rounded	**15**
15	Eyes larger and occupying 1/3 of head side. Antennae shorter, 1/3 of scape length surpassing posterior head corner. Head dorsum finely densely punctate. Dorsa of mesosoma, petiole, and first gastral segment sparsely punctate with interspaces relatively shiny; sides of mesosoma and petiolar node longitudinally rugose, posterior 2/3 of side of first gastral segment smooth and shiny, second gastral segment smooth and shiny. Total length 5–6 mm (India, Philippines, China)	***L.punctiventris* (Mayr)**
–	Eyes smaller and occupying 1/4 of head side. Antennae longer, near 1/2 of scape length surpassing posterior head corner. Head, mesosoma, petiole, and first gastral segment densely punctate with interspaces coarsely reticulate rugose and dull, second gastral segment sparsely punctate. Total length 9–10 mm (Myanmar, India, China)	***L.binghamii* Forel**
16	Clypeus truncate at apex (China)	***L.huapingensis* Zhou**
–	Clypeus convex at apex, not truncate	**17**
17	Dorsal faces of head, mesosoma and petiolar node densely punctate and opaque	**18**
–	Dorsal face of head loosely punctate with interspaces relatively shiny, dorsal faces of mesosoma and petiolar node smooth and shiny	**19**
18	Inner margin of mandible roundly convex, basal corner bluntly angled. Pronotum densely punctate with sides longitudinally striate. Anterodorsal corner of petiolar node broadly rounded in lateral view. Body color black. Robust species with total length 7.1–7.8 mm (China)	***L.zhuangzii* Xu**
–	Inner margin of mandible nearly straight, basal corner rounded without clear angle. Pronotum finely rugulose with sides smooth and shiny. Anterodorsal corner of petiolar node narrowly rounded in lateral view. Body color blackish brown. Slender species with total length 4.5–5.0 mm (China)	***L.laozii* Xu**
19	Petiolar node higher than long in lateral view, with anterodorsal corner narrowly rounded. Mesopleuron and metapleuron mostly smooth and shiny. Body color black to blackish brown. Total length 4.5–5.2 mm (China)	***L.mengzii* Xu**
–	Petiolar node as high as long in lateral view, with anterodorsal corner broadly rounded. Mesopleuron and metapleuron mostly densely rugose and opaque. Body color reddish brown. Total length 5.6–6.4 mm (China)	***L.rufida* Zhou et al.**
20	Petiolar node strongly elongate in lateral view, ~ 1.5 × as long as high (China)	***L.pangui* Xu**
–	Petiolar node moderately to weakly elongate in lateral view, < 1.2 × as long as high	**21**
21	Petiolar node moderately elongate, as long as high or distinctly longer than high in lateral view	**22**
–	Petiolar node weakly elongate, distinctly higher than long in lateral view, 1.3–1.4 × as high as long	**25**
22	Clypeus truncated at apex. Lager species with total length 8–11 mm	**23**
–	Clypeus convex at apex. Smaller species with total length 4.5–7 mm	**24**
23	Clypeus anterior clypeal lobes divided into 2 teeth. dorsal faces between eyes and antennal sockets smooth and shiny, without longitudinal rugae. Petiolar node relatively broader in dorsal view, 1.3 × as long as broad, (India, Sri Lanka, Philippines, China	***L.chinensis* (Mayr)**
–	Clypeus anterior clypeal lobes round, dorsal faces between eyes and antennal sockets longitudinal rugose and opaque. Petiolar node relatively narrower in dorsal view, 2 × as long as broad (Indonesia, Vietnam, Thailand, China)	***L.kraepelini* Forel**
24	Petiolar node distinctly longer than high in lateral view.(PL 0.63, PH 0.51 CASENT0281935) Sides of mesothorax, metathorax, and propodeum mostly smooth and shiny. Body color black. Relatively larger species with total length 5.9–6.3 mm (Vietnam, Myanmar, India, Sri Lanka, Bangladesh, Thailand, Philippines, Singapore, Indonesia, China)	***L.peuqueti* (Andrй)**
–	Petiolar node as high as long in lateral view. Sides of mesothorax, metathorax, and propodeum mostly irregularly rugose and opaque. Body color black, gaster blackish brown. Relatively smaller species with total length 4.5 mm (Japan, China)	***L.confucii* Forel**
25	In full-face view, greatest width of eye roughly equal to or less than the greatest width of antennal scape. Petiolar node relatively longer in lateral view, 1.3 × as high as long, dorsal margin distinctly longer than anterior margin (China)	***L.laeviterga* Zhou et al.**
–	In full-face view, greatest width of eye markedly greater than the maximum width of antennal scape. Petiolar node relatively higher in lateral view, ~ 1.4 × as high as long, dorsal margin as long as anterior margin (China)	***L.sunzii* Xu & He**

## ﻿Discussion

During our specimen collection in Hainan Tropical Rainforest National Park, we found five species of the genus *Leptogenys*: *L.diminuta* (Smith), *L.kitteli* (Mayr), *L.peuqueti* (Andrй), and the two new species. When identifying to which species group *L.hainanensis* belongs, we found that the species *L.amazonica* Borgmeier (CASENT0178836), *L.bohlsi* Emery (CASENT0173510), *L.gatu* Lattke (CASENT0178814), *L.paraensis* Lattke (CASENT0178816), and *L.pubiceps* Emery (CASENT0248759) in the New World *L.unistimulosa* group differed from *L.hainanensis* by having peg-like setae on the clypeal apex and large hypostomal lobes. The species *L.crustosa* Santschi (CASENT0281916) in the Ethiopian *L.conradti* group differed from *L.hainanensis* by the clypeus having a rounded prominent median lobe, but lacking a fringing lamella. The species *L.longensis* Forel (CASENT0217531) in the Australian *L.turneri* group differed from *L.hainanensis* by the clypeus being dentate, whereas *L.hainanensis* has no denticles. However, the characters of the new species described above are the same as those of the species *L.leleji* Zryanin found in Vietnam, so we propose that the two species, *L.hainanensis* and *L.leleji*, be established in a new species group, the *L.leleji* group. The *Leptogenysleleji* group is formally diagnosed as follows: cephalic capsule wider than long; anterior clypeal margin fringed with narrow translucent lamella; mandibles linear, a large gap formed between clypeus and mandible when fully closed; basal flagellar (third antennal) segment elongate; dorsum of body with standing hairs; propodeum with lateral teeth, posterior apex of petiole in profile drawn out into a tooth. A formal diagnosis for this species group awaits further revisions of Asian *Leptogenys*.

In the island ecosystem of China’s Hainan Province, a plethora of hitherto undiscovered species awaits exploration. A comprehensive future survey promises to augment the existing inventory of ant species in China. An unpublished study employing both sample-plot and search-collecting methods has revealed a total of 72 ant species in Hainan, including the two new species- described here. Regrettably, due to constraints in collection methods, the number of specimens retrieved for the two new species was limited. Both species, *L.hainanensis* and *L.zhoui*, were collected while foraging along streams in tropical rainforests at elevations of less than 1,000 meters. The individuals of our recent taxonomic discoveries were procured during foraging excursions, precluding the identification of their nests. Subsequent iterative surveys should yield more specimens, thus substantiating the status of these species.

## Supplementary Material

XML Treatment for Leptogenys hainanensis

XML Treatment for Leptogenys zhoui

## ﻿Additional information

### Author ORCIDs

Chao Chen https://orcid.org/0009-0007-2983-9570

Zhilin Chen https://orcid.org/0000-0001-6564-1528

Zhenghui Xu https://orcid.org/0000-0003-2415-770X

Qishan Fu https://orcid.org/0009-0009-5378-6127

Liwen Fu https://orcid.org/0009-0008-2319-3593

### Data availability

All of the data that support the findings of this study are available in the main text or Supplementary Information.

## Supplementary materials

Supplementary material 1 Habitat photos of two new species Data type pdf https://binary.pensoft.net/file/1002202This dataset is made available under the Open Database License (http://opendatacommons.org/licenses/odbl/1.0/). The Open Database License (ODbL) is a license agreement intended to allow users to freely share, modify, and use this Dataset while maintaining this same freedom for others, provided that the original source and author(s) are credited.Chao Chen, Zhilin Chen, Zhenghui Xu, Qishan Fu, Liwen Fu

Supplementary material 2 Specific geographical coordinates of the two new species Data type csv https://binary.pensoft.net/file/1002203This dataset is made available under the Open Database License (http://opendatacommons.org/licenses/odbl/1.0/). The Open Database License (ODbL) is a license agreement intended to allow users to freely share, modify, and use this Dataset while maintaining this same freedom for others, provided that the original source and author(s) are credited.Chao Chen, Zhilin Chen, Zhenghui Xu, Qishan Fu, Liwen Fu
