# Supplementary material for: ﻿Two new ant species of the genus Leptogenys (Hymenoptera, Formicidae) from Hainan, China, with a key to the known Chinese species
Source: Zookeys. 2024 Mar 15;1195:199–217. doi: 10.3897/zookeys.1195.115889 (PMC10960152; doi:10.3897/zookeys.1195.115889)
Supplement: Supplementary material 1 — Habitat photos of two new species [file zookeys-1195-199_article-115889__-s001.pdf]

habitat photos

***Leptogenys hainanensis* Chen, Chen & Xu, sp. nov.**

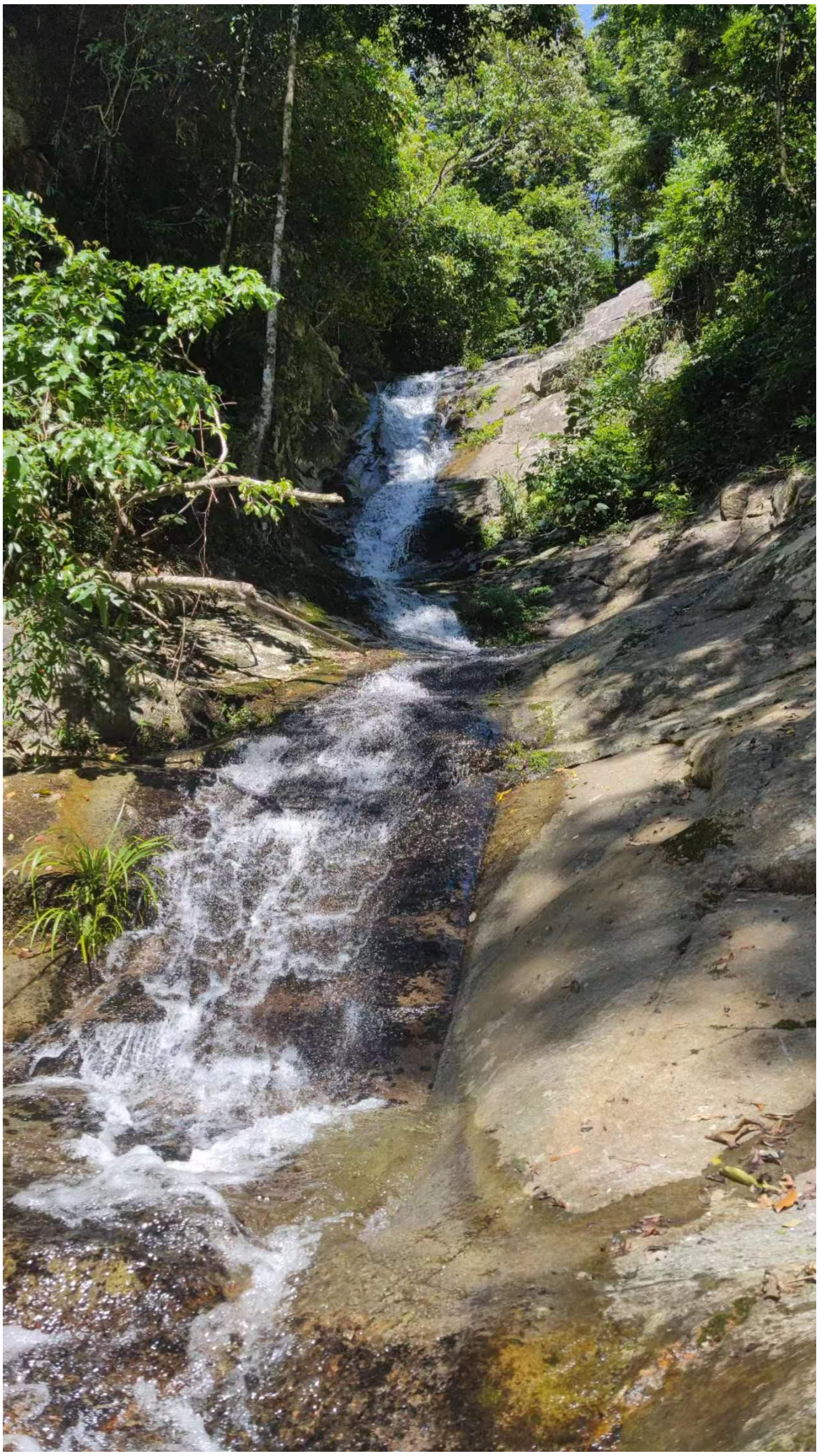

Hainan Province, Qiongzhong County, Yinggeling Nature Reserve, Yinggezui sub-station, 19°02′54″N, 109°33′33″E, 750m

Leptogenys zhoui **Chen, Chen & Xu, sp. nov.**

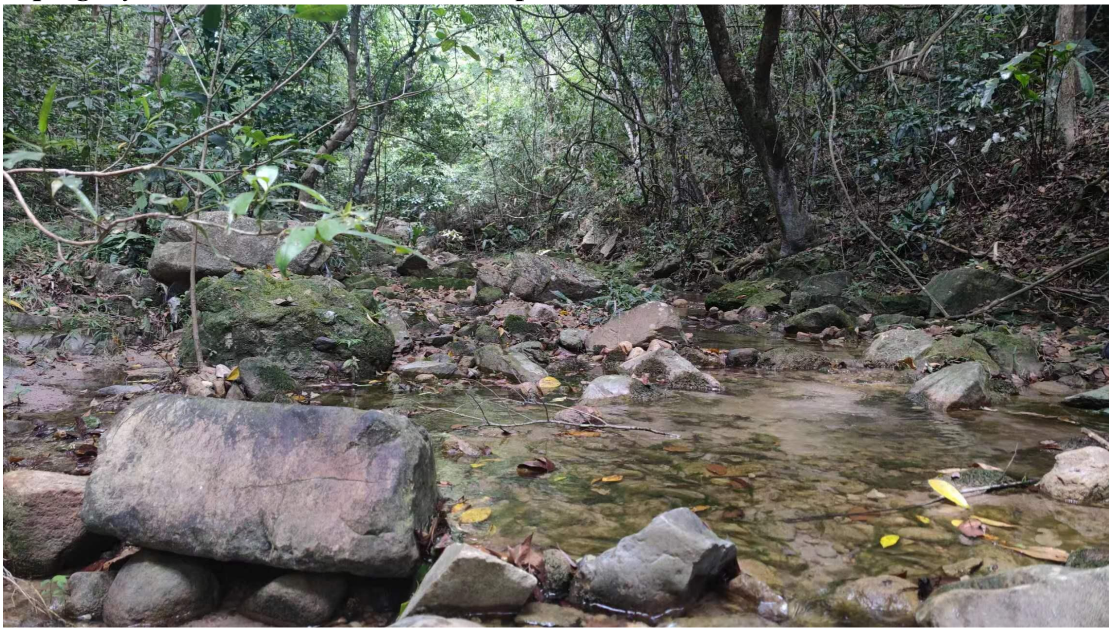

Hainan Province, Baisha County, Nanmeiling forest park, Yaqiong sub-station, 19°08′39″N, 109°20′57″E, 700m
